# Ginsenoside Rg1 Improves Inflammation and Autophagy of the Pancreas and Spleen in Streptozotocin-Induced Type 1 Diabetic Mice

**DOI:** 10.1155/2023/3595992

**Published:** 2023-03-14

**Authors:** Yi Zong, Weihua Yu, Hanghang Hong, Zhiqiang Zhu, Wenbo Xiao, Kewu Wang, Guoqiang Xu

**Affiliations:** ^1^Department of Radiology, The Fourth Affiliated Hospital, Zhejiang University School of Medicine, Yiwu, China; ^2^Department of Gastroenterology, The Fourth Affiliated Hospital, Zhejiang University School of Medicine, Yiwu, China; ^3^Department of Ultrasound, The Fourth Affiliated Hospital, Zhejiang University School of Medicine, Yiwu, China; ^4^Department of Clinical Laboratary, The Fourth Affiliated Hospital, Zhejiang University School of Medicine, Yiwu, China; ^5^Department of Radiology, First Affiliated Hospital, School of Medicine, Zhejiang University, Hangzhou, China; ^6^Department of Gastroenterology, First Affiliated Hospital, School of Medicine, Zhejiang University, Hangzhou, China

## Abstract

**Background:**

Ginsenoside Rg1 (Rg1) is one of the key bioactive components of the precious Traditional Chinese Medicine that has been used to treat diabetes in China. Ginsenosides have been reported to protect diabetics from tissue damage, inflammation, and insulin resistance. Type 1 diabetes (T1D) is an organ-specific autoimmune disease that occurred frequently among adolescents over the world, its development was related to inflammation and *β*-cells immunodeficiency. The aim of this study is to explore the biological mechanism of Rg1 on inflammation and autophagy of *β*-cells in T1D and its therapeutic potential.

**Methods:**

The model of T1D mice was established by injecting Streptozotocin (STZ) (55 mg/kg) or citric acids once a day for 5 days and from the fourth day of injection, mice were administered with Rg1 (20 mg/kg) or saline by gavage once a day for 12 days. Hematoxylin-eosin staining, immunofluorescence, ELISA, quantitative real-time PCR, and Western blot were used to observe the histopathological changes, inflammatory factor levels, and autophagy markers after administration of ginsenoside Rg1.

**Results:**

Compared to the T1D mice, Rg1 improved the weight (*p* < 0.05) and blood glucose (*p* < 0.01) of mice, advanced the injury and apoptosis of *β*-cells in islets (*p* < 0.01), and markedly inhibited the protein expression degrees of CD45, CXCL16, ox-LDL, and TF in the pancreas and spleens (*p* < 0.01), also activated the degrees of insulin in serum (*p* < 0.01). Besides, in T1D mice' pancreas and spleen, Rg1 markedly repressed the IL-1*β*, TNF-*α*, and NOS2 mRNA levels (*p* < 0.05 or *p* < 0.01), inhibited the CXCL16, NF-*κ*B, and TF proteins (*p* < 0.05 or *p* < 0.01), while elevating the ratio of LC3 II/I (*p* < 0.01) and P62 (*p* < 0.05) protein level.

**Conclusions:**

This study proved that Rg1 protected mice against T1D possibly by improving islet injury and tissue inflammation, raising serum insulin, and tissue autophagy marker.

## 1. Introduction

Type 1 diabetes (T1D) is an organ-specific autoimmune disease with selective destruction of *β*-cells in islets and dysfunction of insulin secretion, affecting more than 490 thousand of the world's children [1]. Epidemiological studies suggest that childhood obesity, eating habits, virus infection, and other environmental factors are related to the onset of T1D [2]. Additionally, the destruction of the *β*-cell is one of the reasons to result in the reduction of insulin, the uncontrolled production of glucose, and hyperglycemia [3]. Inhibition of hyperglycemia has always been the focus of T1D treatment [4]. Additionally, modern science focused on preventing or postponing *β*-cell loss in T1D [5, 6]. Scientists reported that the autoimmune response in T1D resulted in an inflammatory state in *β*-cells [7]. Clinical evaluation of children with high genetic risk found that inflammation, cytotoxicity, angiogenesis, and antigen-presenting cell activity increased in children progressing towards islet autoimmunity [8]. Also, Park's team noticed that Alpha-1 antitrypsin with anti-inflammatory properties favorably impacted the development of T1D in mice [9]. It suggests that the anti-inflammatory treatment of T1D may be effective.

Scientists found that many plant extracts have the potential to improve diabetes [10–12]. Extracts of *Olea europaea* treatment reduced fasting blood glucose in diabetes animals [13]. Scientists reported that the extract of *Malva neglecta* Wallr inhibited the level of oxidative stress in diabetes animals [14, 15]. Ginsenoside Rg1 (Rg1) is one of the key bioactive components of *Panax ginseng C. A. Mey* (family *Araliaceae*), a precious Traditional Chinese Medicine that has been used to treat diabetes in China [16, 17]. Rg1 is a triterpenoid saponin containing a protopanaxatriol structure [18]. In recent years, scientists have discovered that ginsenosides can protect tissues such as the heart, pancreas, and spleen, antagonize inflammation, and improve insulin resistance in diabetes [19–21]. Research pointed out that Rg1 inhibited the IL-1*β* and IL-18 levels in T1D mice [22]. Similarly, Yu and colleagues studied that Rg1 could prevent the high glucose-/palmitate-induced damage in H9C2 cells via the AKT-GSK-3beta-Nrf2 pathway [23]. And Luo and colleagues reported that Rg1 blocked the pro-inflammatory effects of lipopolysaccharide on neonatal rat cardiomyocytes [24]. In addition, the regulation of autophagy in *β*-cells is important to maintain the stability of insulin [25]. A study reported that inhibiting autophagy promoted the IL-1*β* level [26]. And extracts of ginsenoside can induce protective autophagy in HepG2 cells [27] and regulate autophagy in acute liver injury [28]. Modern research argues that inflammation and autophagy play crucial roles in the treatment of diabetes [29, 30]. However, there was still a lack of research on the biological effect of Rg1 on T1D. To explore the biological mechanism of Rg1 on inflammation in T1D and study its therapeutic potential, this study constructed the T1D mouse model by injecting STZ and proposed the possibility that Rg1 ameliorates inflammation in T1D mice via elevating autophagy.

## 2. Materials and Methods

### 2.1. Animals and Treatments

**Figure 1:**
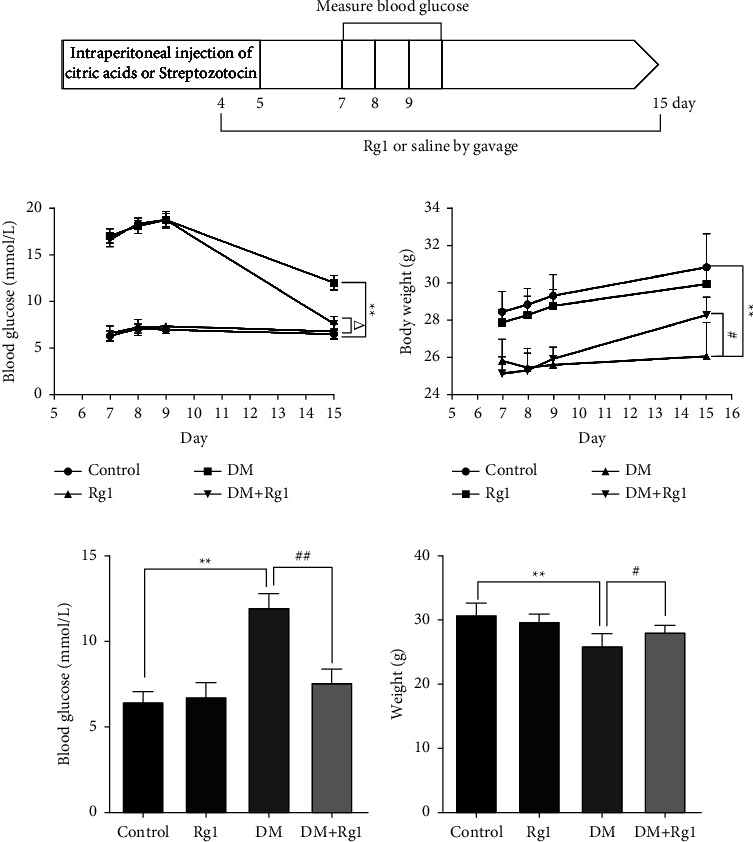
Effects of ginsenoside Rg1 (Rg1) on blood glucose and body weight in type 1 diabetes (T1D) mice. (a) The time axis of animal treatment. (b, c) Changes in blood glucose and body weight on days 7, 8, 9, and 15 since the first injection of streptozotocin (SZT). (d, e) The statistical analysis of blood glucose and body weight in different groups on days 15 since the first injection of SZT. Those results suggested the T1D mouse models were successfully constructed, and Rg1 treatment improved blood glucose and body weight in T1D. (Mean ± SD, *n* = 6) ^*∗∗*^*p* < 0.01, vs. the control group; ^Δ^*p* < 0.05, vs. the Rg1 group; ^#^*p* < 0.05, ^##^*p* < 0.01 vs. the DM group.

Male C57BL/6 mice (25–30 g) aged seven to eight weeks were provided by Shanghai Ling Chang Biotech Co., Ltd. (Shanghai, CHN). All animal tests were performed according to the guidelines of the Institutional Animal Care and Use Committee and approved by the Animal Experimentation Ethics Committee of the Zhejiang Eyong Pharmaceutical Research and Development Center (Certificate No. SYXK (Zhe)2021-0033). A temperature-, humidity-, and light-controlled animal room (20°C, 60% humidity, and a 12 h light/dark cycle) was used to house all mice with free access to food and water for 7 days. The mice were divided into 4 groups with 6 mice in each group: the mice in the DM group and the DM + Rg1 group were injected intraperitoneally with Streptozotocin (STZ) (55 mg/kg) (98%, S817944, Mackin, Shanghai, CHN) that was dissolved in citric acids (CA, 50 mM, pH4.5) once a day for 5 days; the mice in the control group and Rg1 group were injected intraperitoneally with CA for 5 days, whose volume was equal to the amount of STZ in the DM group. Additionally, the mice in the Rg1 group and DM + Rg1 group were further administered with Rg1 (20 mg/kg) (≥98%, G909436, Mackin, Shanghai, CHN) by gavage once a day for 12 days since the fourth day of STZ/CA injection. The time axis of animal treatments is shown in Figure 1(a). The dose of Rg1 was set according to previous studies on Rg1 [22, 31].

### 2.2. Evaluation of Type 1 Diabetic Mice Modeling

On the 7th day after the last STZ was injected, the tail vein blood of mice was randomly taken to measure blood glucose for 3 days, and the weight of the mice was recorded. The level of blood glucose was greater than or equal to 16.7 mmol/L for 3 consecutive days, indicating the success of the T1D model.

### 2.3. Collection of Serum and Tissue Samples

On the 10^th^ day after the last injection of STZ, the mice were anesthetized with ether and euthanasia with carbon dioxide. The pancreas and spleen were collected and kept in 10% saline formalin. The serum was separated by centrifugation at 4000 rpm for 15 min from the blood that was collected from the retroorbital venous plexus by small capillary tubes before euthanasia. Tissue and serum were stored at −80°C for subsequent experiments.

### 2.4. Detection of ox-LDL and Insulin (INS) in Serum

According to the instructions of the ox-LDL (MM-0908M1, Meimian, Jiangsu, CHN) and INS (MM-0579M1, Meimian, Jiangsu, CHN) ELISA kits, serum samples were added to the enzyme label plate to incubate at 37°C for 60 min. After washing the plate, chromogenic agents A and B that were added to the plate were well-mixed and stood in the dark for 15 min, before being finally added to be termination solution to terminate the reaction. The absorbance (expressed as an OD value) was detected at a wavelength of 450 nm, and there were 6 parallel setups in each group.

### 2.5. Hematoxylin-Eosin (HE) Staining of the Pancreas

Pancreases have been fixed, dehydrated with an alcohol gradient, made transparent with xylene and alcohol, and then embedded in paraffin. The tissues with paraffin wrapping were made into 5 *μ*m sections and stained by HE staining kits (G1003, ServiceBio, Wuhan, CHN). An optical microscope was used to observe the tissues. The histology of the pancreas was evaluated according to the islet volume and the regular edges of the islet, and the score was proportional to the degree of injury.

### 2.6. Immunofluorescence of CXCL16, CD45, Insulin Receptor (INSR), ox-LDL, and Tissue Factor (TF) in the Pancreas and Spleen

The sections were repaired with an EDTA antigen repair buffer (pH = 8, G1206, Servicebio, CHN) in a microwave, and the 3% BSA was added dropwise to block for 30 min. Then the CD45R (1 : 50, sc19597, Santa, US), INSR-*β* antibody (1 : 50, sc57342, Santa, US), CXCL16 (1 : 100, DF13312, Affinity, CHN), anti-LDL receptor antibody (1 : 100, ab30532, Abcam, US) and anti-TF antibody (F-8) (1 : 50, sc-373785, SCBS, US) were used for incubating with the sections overnight at 4°C, and then after washing, the tissue that was incubated with the goat antirabbit IgG H&L (ab150078, Abcam, US) that was incubated in dark for 50 min. Following PBS washing, the nuclei were counterstained with DAPI (G1012, ServiceBio, CHN). Thirdly, after being washed, the sections were quenched by the antifluorescence quenching sealing reagent (G1401, Servicebio, CHN) for 5 min and rinsed with running water for 10 min. Between every two steps, the sections were washed 3 times with PBS (pH = 7.4) on the shaking table. Finally, a NIKON eclipse upright microscope was used to observe and image the fluorescence (the nucleus is blue, and the positive expression is red or green).

### 2.7. Quantitative Real-Time PCR Analysis (qRT-PCR) of Inflammatory Factors in the Pancreas and Spleen

The tissue was lysed with the Total RNA Extractor (B511311, Sangon Biotech, CHN) and centrifuged to remove the precipitation, then the supernatant was dissolved in chloroform and centrifuged again to separate the supernatant. The supernatant was soaked in isopropanol for 15 minutes to obtain precipitation, and then the precipitation was soaked in 75% absolute ethanol. Finally, the dried precipitate was dissolved in DEPC water and subjected to reverse a transcription reaction by HiFiScript cDNA Synthesis Kit (CW2569, CWBIO, CHN). The qRT-PCR was carried out according to the following procedure: denaturation at 95°C for 10 min and 95°C for 15 s, then at 60°C for 60 s, performing 40 cycles. Primer information is shown in Table 1.

### 2.8. Western Blot of CXCL16, NF-*κ*B/P65, TF, LC3, and P62 in the Pancreas and Spleen

The tissue was soaked in RIPA lysate and homogenized on ice; then it was centrifuged. The total protein concentration was measured by the BCA Protein Assay Kit (PC0020, Solarbio, CHN) and mixed with a loading buffer (1 : 4) in a 100°C water bath for 5 min. The SDS-PAGE and PVDF membranes were used to separate the protein by vertical electrophoresis and wet transfer. Following blocking by 5% BSA, the sample was incubated with the primary antibody for one night. Secondarily, the sample was incubated with HRP-conjugated goat IgG secondary antibodies at 20°C for 1 h. After wet transfer, the sample was cleaned 3 times/10 min with TBST between every two steps. Finally, ECL luminescent reagent was used to present the photo in the chemiluminescence imager. The antibodies for CXCL16 (1 : 2000, DF13312) and GAPDH (1 : 5000, AF7021) were purchased from Affinity Biosciences, Ltd. (Jiangsu, CHN). And NF-*κ*B (1 : 1000, 6956T), LC3 (1 : 1000, 4599T), and P62 (1 : 1000, 5114T) antibodies were provided by Cell Signaling Technology, Inc. (MA, US). The TF antibody (1 : 100, sc-373785) was bought from Santa Cruz Biotechnology, CA (MO, US).

### 2.9. Statistical Analyses

Data analysis by SPSS 16.0. The comparisons of multiple groups were analyzed by one-way ANOVA followed by Tukey, and the *t*-test was used to analyze the differences between every two groups. If it is a multigroup comparison with a nonnormal distribution or uneven variance, the Kruskal−Wallis *H*-Test was used. All data were expressed as mean ± standard deviation (mean ± SD), *p* < 0.05 meaning that the difference was statistically significant.

## 3. Results

### 3.1. Rg1 Improved the Blood Glucose and Body Weight in T1D Mice

On day 7 after the first intraperitoneal injection of STZ, the blood glucose of mice ≥16.7 lasted for 3 days, which means that the T1D models in the DM group and the DM + Rg1 group were successfully constructed (Figure 1(b)). At the end of the 15th day of Rg1 gavage, the blood glucose in the DM group was remarkably higher than that in the control group. While the blood glucose in the DM + Rg1 group was remarkably lower than that in the DM group (Figures 1(b) and 1(d)). The weight of mice in the DM group was lower than that in the control group, while the weight of mice in the DM + Rg1 group was higher than that in the DM group on day 15 (Figures 1(c) and 1(e)).

### 3.2. Rg1 Ameliorated the Pathological Damage to the Pancreas in T1D Mice

**Figure 2:**
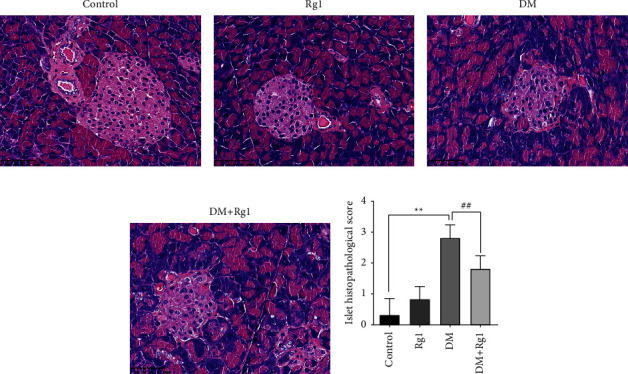
Hematoxylin-eosin (HE) staining and the histopathological score of the pancreas suggested Rg1 treatment on type 1 diabetes mice improve the histopathological injury of pancreas. (a–d) Histopathological picture of HE stains. (×400, scale: 50 *μ*m). (e) The islet histopathological score made by technicians who didn't know the attribution of pancreatic tissues (mean ± SD, *n* = 6). The histology of the pancreas was evaluated according to the islet volume and the regular edges of the islet, and the score was proportional to the degree of injury. ^*∗∗*^*p* < 0.01, vs. the control group; ^##^*p* < 0.01 vs. the DM group.

The type 1 diabetic mouse model was built successfully, as shown in Figure 2, the results of pancreatic HE staining. In the control and Rg1 groups, the islet was plump, and the edges of islet cells were evenly regular (Figures 2(a) and 2(b)). While in the DM group, the size and edges of the islets were uneven (Figure 2(c)). And Rg1 treatment could improve the histology of the injury (Figure 2(d)). Compared with the control group, the score of the DM group was notably higher (*p* < 0.01). And the score of the DM + Rg1 group was immensely lower (Figure 2(e)).

### 3.3. Rg1 Regulated the Expressions of CXCL16, CD45, and INSR in the Pancreas

**Figure 3:**
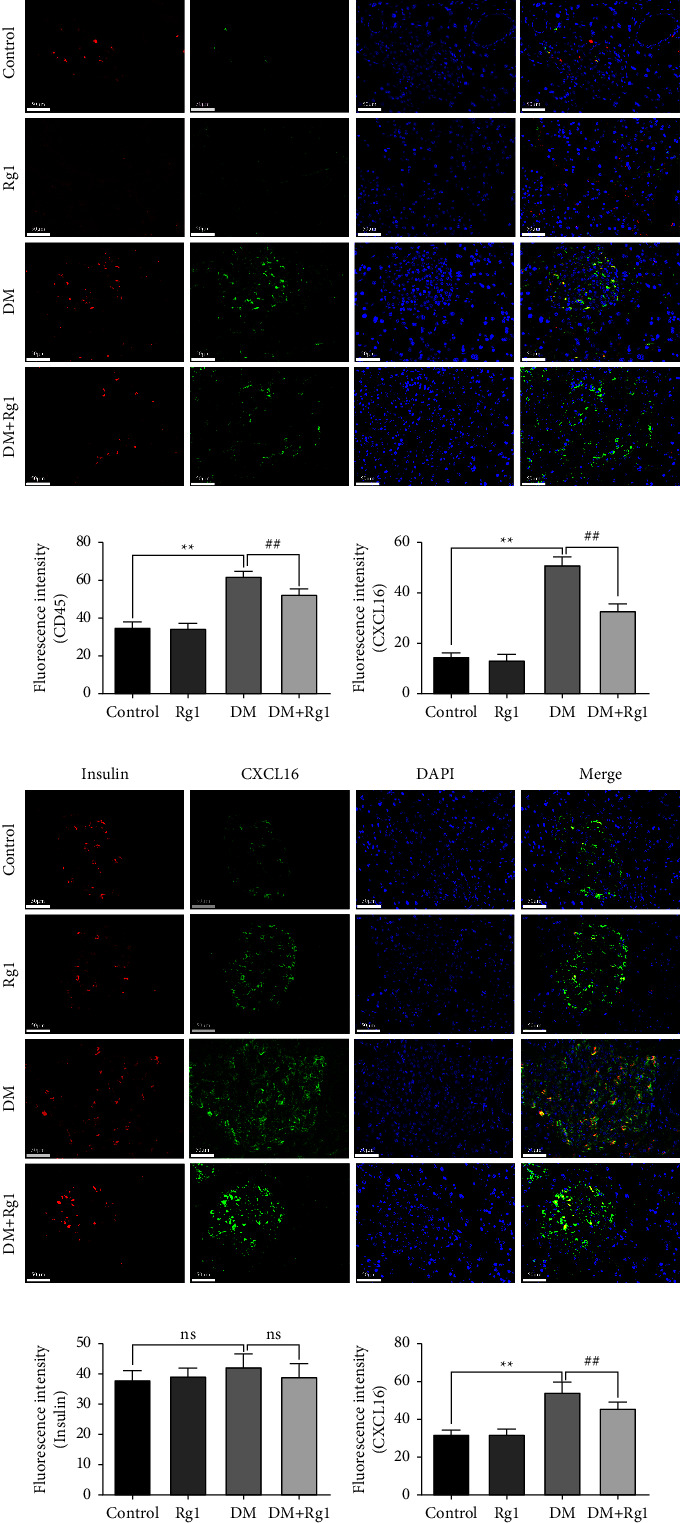
Expressions of CXCL16, CD45, and insulin receptor (INSR) proteins in the pancreas by immunofluorescence (×400, scale: 50 *μ*m, mean ± SD, *n* = 6) (a–c) the expressions of CXCL16 and CD45 proteins' colocalization in the pancreas. (d–f) The expressions of INSR and CXCL16 proteins colocalization in the pancreas. The fluorescence intensity was detected to observe the protein expression. The increase in CD45 indicated an increase in inflammatory cell infiltration. Those results suggested that Rg1 treatment improves inflammation of the pancreas in type 1 diabetes mice relating to reducing CXCL16. ^*∗∗*^*p* < 0.01, vs. the control group; ^##^*p* < 0.01 vs. the DM group.

The colocalization of CD45 or INSR protein with CXCL16 in the pancreas was observed by immunofluorescence (Figures 3(a)–3(f)). The CD45 positive expression was obviously detected in the DM group, which was notably higher than that in the control group (*p* < 0.01), and the expression of the colocated CXCL16 was also higher in the DM group too (*p* < 0.01). Then it was obvious that the expressions of CD45 and CXCL16 proteins decreased enormously by Rg1 treatment in DM mice (*p* < 0.01) (Figures 3(b) and 3(c)). By analyzing the level of INSR expression, it was found that there were no statistical differences in it among the four groups (*p* > 0.05). Then the expression of CXCL16 was significantly upregulated in the DM group compared to the control group, while the CXCL16 expression was significantly reduced by Rg1 treatment (*p* < 0.01) (Figures 3(d)–3(f)).

### 3.4. Rg1 Regulates the Expression of CXCL16 and CD45 Proteins in the Spleen

**Figure 4:**
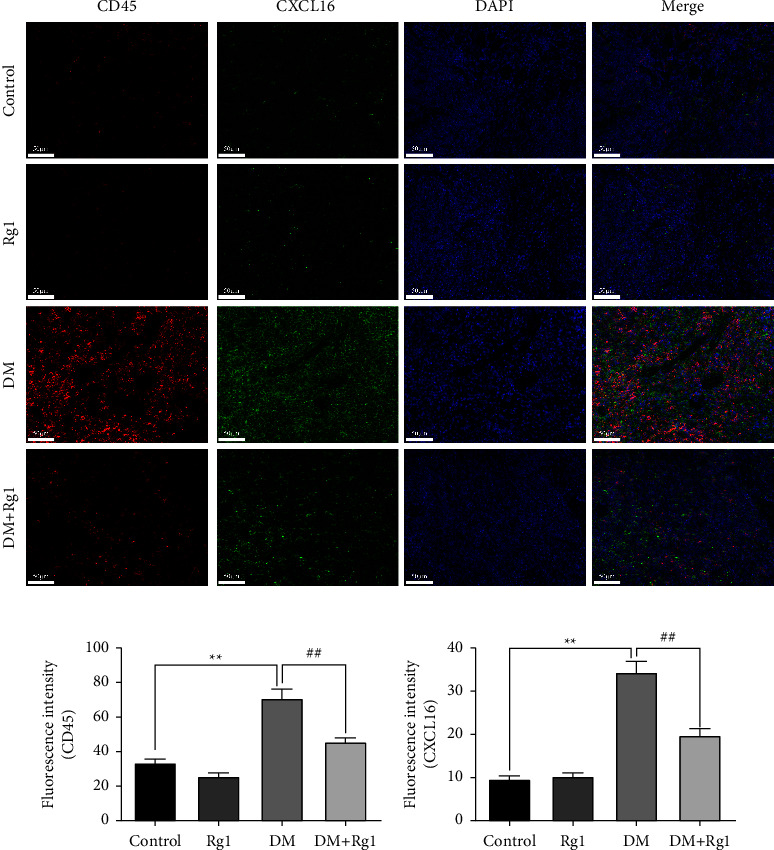
The CXCL16 and CD45 proteins are expressed in the spleen through immunofluorescence. (×400, Scale: 50 *μ*m) (a–c) the expressions of CXCL16 and CD45 protein colocalization in the spleen. (Mean ± SD, *n* = 6) The fluorescence intensity was detected to observe the protein expression. The increase in CD45 indicated an increase in inflammatory cell infiltration. Those results suggested Rg1 treatment improve inflammation of the spleen in type 1 diabetes mice relating to reducing CXCL16. ^*∗∗*^*p* < 0.01, vs. the control group; ^##^*p* < 0.01 vs. the DM group.

Through an immunofluorescence assay, we observed the colocalization of CXCL16 and CD45 in the spleen (Figures 4(a)–4(c)). The levels of CD45 and CXCL16 proteins were markedly increased in the DM group compared to the control group (*p* < 0.01). While in the DM + Rg1 group, the levels of CD45 and CXCL16 were both lower than that in the DM group (*p* < 0.01).

### 3.5. Rg1 Regulates the Expression of ox-LDL an TF Proteins in the Pancreas and Spleen

**Figure 5:**
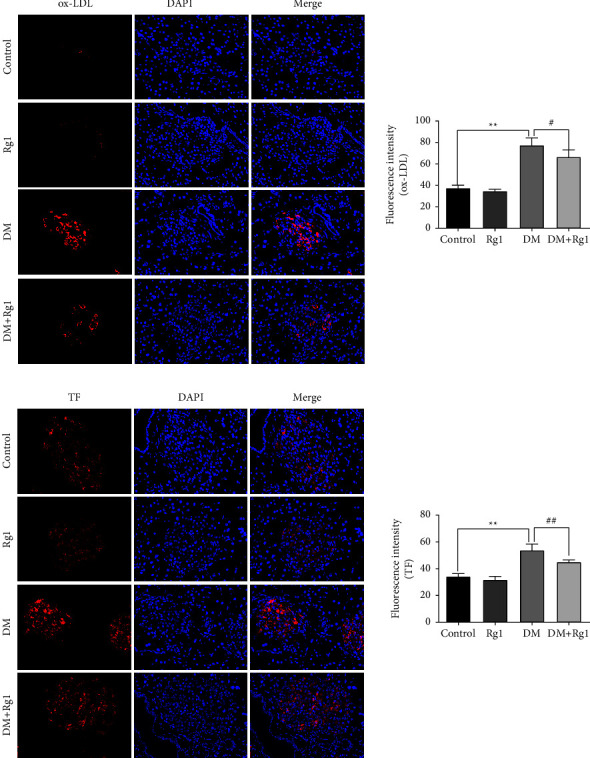
The ox-LDL and TF proteins are expression in the pancreas through immunofluorescence. (×400, Scale: 50 *μ*m) (a) the expression of ox-LDL protein in the pancreas. (b) The expression of TF protein in the pancreas. The fluorescence intensity was detected to observe the protein expression. These results suggest the improvement of Rg1 on the pancreas of type 1 diabetes mice may be by inhibiting the ox-LDL and TF levels. (Mean ± SD, *n* = 6) ^*∗∗*^*p* < 0.01, vs. the control group; ^#^*p* < 0.05, ^##^*p* < 0.01 vs. the DM group.

**Figure 6:**
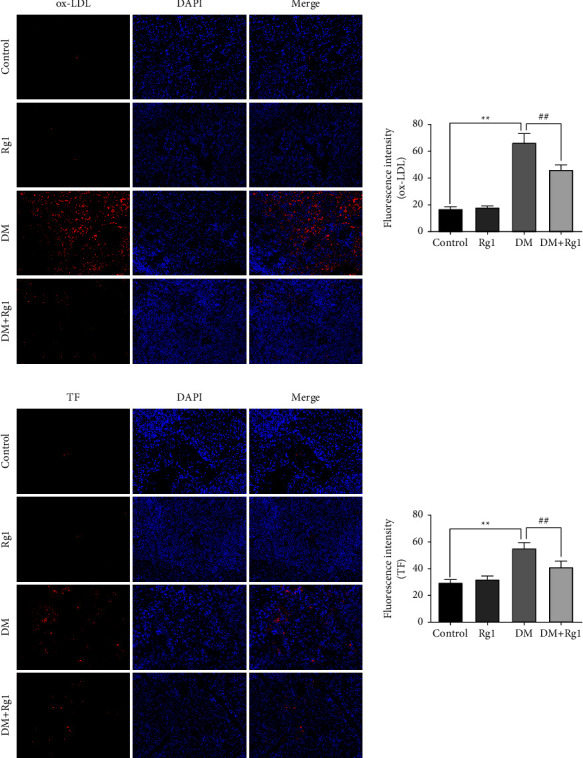
The ox-LDL and TF proteins are expressed in the spleen through immunofluorescence. (×400, Scale: 50 *μ*m; mean ± SD; *n* = 6) (a) the expression of ox-LDL protein in the spleen. (b) The expression of TF protein in the pancreas. The fluorescence intensity was detected to observe the protein expression. These results suggest the improvement of Rg1 on the spleen of type 1 diabetes mice may be caused by inhibiting the ox-LDL and TF levels. ^*∗∗*^*p* < 0.01, vs. the control group; ^##^*p* < 0.01 vs. the DM group.

The degrees of ox-LDL and TF proteins in the pancreas and spleen of T1D mice were observed by immunofluorescence (Figures 5 and 6). In the pancreas and spleen, compared with the control group, the degrees of ox-LDL and TF proteins in DM mice in the Rg1 group were not significant (*p* > 0.05), the expression of these two proteins in the DM group was significantly upregulated (*p* < 0.01). The ox-LDL and TF proteins levels of the pancreas and spleen in the DM + Rg1 group compared with the DM group was markedly reduced (*p* < 0.01 and *p* < 0.05).

### 3.6. Rg1 Inhibited the Level of ox-LDL and Advanced the Degree of INS in Serum

**Figure 7:**
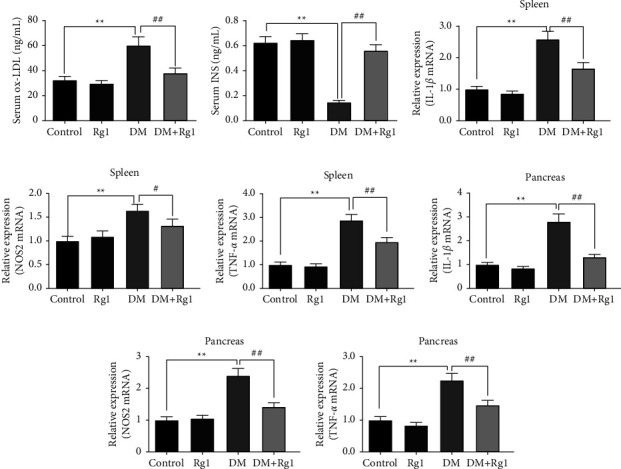
The levels of ox-LDL and INS in the serum and the transcription levels of inflammatory factors in the pancreas and spleen. (Mean ± SD) (a) The levels of ox-LDL and (b) the degrees of INS in serum were detected by ELISA (*n* = 6). (c–e) The transcription level of inflammatory factors in the spleen was detected by qRT-PCR (*n* = 3). (f–h) The transcription level of inflammatory factors in the pancreas was detected by qRT-PCR (*n* = 3). The way of 2^−△△Ct^ was used to calculate the relative expression of the target gene. Rg1 treatment in type 1 diabetes mice can inhibit serum ox-LDL and the transcription levels of inflammatory factors in the pancreas and spleen and advance the serum INS. ^*∗∗*^*p* < 0.01, vs. the control group; ns *p* > 0.05, ^##^*p* < 0.01 vs. the DM group.

As Figures 7(a) and 7(b) show, according to the results of the ELISA, we noticed that Rg1 treatment inhibited the levels of ox-LDL and advanced the expression degree of INS in the serum of T1DM. In the DM group, the level of ox-LDL was immensely upregulated (*p* < 0.01), and the INS level was repressed markedly more than that in the control group (*p* < 0.01). While in the DM + Rg1 group, compared with the DM group, the degree of ox-LDL was immensely inhibited (*p* < 0.05), and the INS level was significantly increased (*p* < 0.01).

### 3.7. Rg1 Restrained the Transcription Level of Inflammatory Factors in the Pancreas and Spleens

The transcription degrees of IL-1*β*, TNF-*α*, and NOS2 were semiquantified by qRT-PCR. The results are shown in Figures 7(c)–7(h), in the DM group, the degrees of IL-1*β*, TNF-*α*, and NOS2 mRNA in the pancreas and spleen were notably superior to those in the control group (*p* < 0.01). And in the DM + Rg1 group, the transcription degrees of IL-1*β*, TNF-*α*, and NOS2 in the pancreas and spleens were inhibited compared with those in the DM group (*p* < 0.05) (Figures 7(c) and 7(h)).

### 3.8. Rg1 Suppresses the Expression of CXCL16, NF-*κ*B, TF, and Activated LC3 and P62 Proteins in the Pancreas and Spleen of DM Mice

**Figure 8:**
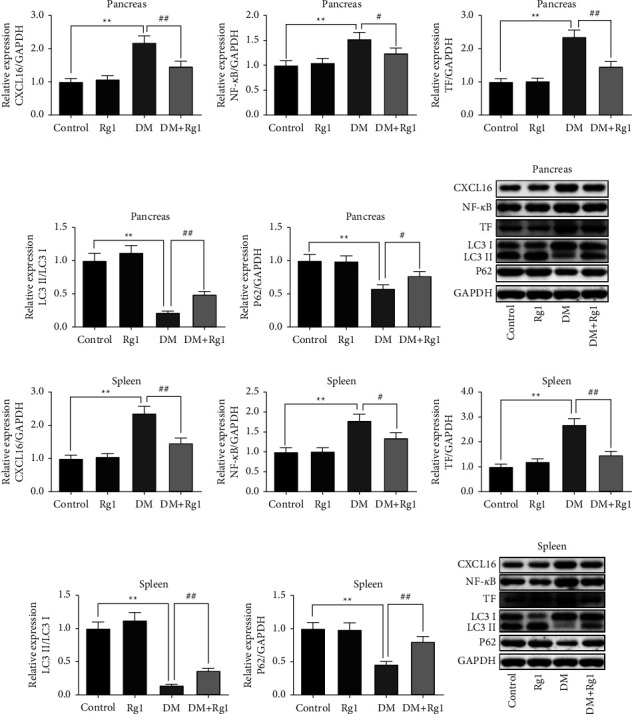
The expression of CXCL16, NF-*κ*B, TF, LC3, and P62 proteins in the pancreas and spleens. (Mean ± SD, *n* = 3) (a–f) the expression of CXCL16, NF-*κ*B, TF, LC3, and P62 proteins in the pancreas. (g–l) The expression of CXCL16, NF-*κ*B, TF, LC3, and P62 proteins in the spleens. The improvement of Rg1 treatment on type 1 diabetes mice was related to the increase of CXCL16, NF-*κ*B, and TF and the raised autophagy (IL3 II/LC 3 I). ^*∗∗*^*p* < 0.01, vs. the control group; ns *p* > 0.05, ^##^*p* < 0.01 vs. the DM group.

By WB analyzing, in the DM group, the expression of CXCL16, NF-*κ*B, and TF proteins in the pancreas and spleen were raised prominently compared to the control group (*p* < 0.01), and the degrees of CXCL16, NF-*κ*B, and TF proteins were also huge down-regulated in the Rg1 treatment T1DM mice (*p* < 0.05) (Figures 8(a)–8(c) and 8(g)–8(i)).

On the contrary, the LC3 and P62 protein levels in the DM group were inhibited significantly compared with the control group (*p* < 0.01) (Figures 8(d), 8(e), 8(j) and 8(k)). And compared with the DM group, the expression degrees of LC3 protein in the pancreas and spleen were elevated enormously in the Rg1 treatment T1DM mice (*p* < 0.01) (Figures 8(d) and 8(j)). As well as the level of P62 protein in the pancreas and spleen was raised markedly in the DM + Rg1 group (*p* < 0.05) (Figures 8(e) and 8(k)).

## 4. Discussion

The *β*-cells in islets are the main sites to regulate INS. Islet atrophy and apoptosis of *β*-cells in T1D rats is increased. Research reported that improved the apoptosis of *β*-cells and the morphology of islets were able to reduce blood glucose levels in the model rats [32]. This report discovered that Rg1 treatment on DM mice advanced the mice's weight, improved the number and morphology of islets, and inhibited the blood glucose level. Also, Rg1 treatment raised the serum INS level. It reminded that Rg1 can improve the function of the islet in T1D mice. Besides, a piece of evidence displayed an increased ox-LDL level in T1DM patients [33]. The increase of ox-LDL induced lipid accumulation and an inflammatory response, which caused damage to tissues [34, 35]. And activation of ox-LDL could induce the inflammatory response and promote the secretion of inflammatory factors, such as TF, the inflammatory biomarker, which would be activated through the CXCL16/ox-LDL pathway in *β*-cell [36, 37]. CXCL16, a special chemokine, can combine with immune cells to drive into the inflammatory part, and some can internalize ox-LDL [38, 39]. This study observed the colocalized expression of INSR or CD45 with CXCL16 by immunofluorescence and found there was inflammatory infiltration in the pancreas of T1D mice, and Rg1 treatment could significantly improve this situation. The above experimental results indicated that Rg1 had the potential to improve T1D mice by adjusting ox-LDL.

Furthermore, this study found IL-1*β* and TNF-*α* levels were repressed significantly by Rg1 treatment in the pancreas of DM model mice. A study reported that in macrophages of diabetics, the IL-1*β* and TNF-*α* levels increased significantly [40–42]. Additionally, scientists have proved that *β*-cell injury in T1D was accompanied by the up-regulation of inflammatory factors, NF-*κ*B and iNOS, while the expression levels of them were decreased after improving the injury [43]. Dampening NF-*κ*B-iNOS-NO pathway negatively regulated the inflammation and apoptosis of *β*-cells [44]. Through qRT-PCR and WB, this study found the expression of NF-*κ*B and iNOS was antagonized by Rg1 treatment. In short, it is suggested that Rg1 can antagonize the inflammation of the pancreas in T1D mice via inhibiting the NF-*κ*B-iNOS signaling pathways.

Additionally, scientists have proven that immune deficiency in diabetes is associated with the damage of the spleen [45]. Moreover, splenocytes from a diabetic animal can transfer the T1D to healthy recipients [46]. Therefore, this study focused on the effect of Rg1 on the spleen of T1D mice and found it could improve the inflammation in the spleen. Studies have reported IL-1*β*, TNF-*α*, and NOS2 were closely related to macrophages polarization and macrophages played an important role in the development of inflammation [47]. The results of this study indicated that the levels of macrophage polarization-related cytokines were enormously upregulated in the spleen of T1D mice, and Rg1 treatment was able to reduce them in the spleen. Rg1 treatment could significantly inhibit macrophage polarization and improve inflammation in the spleen of T1D mice.

Moreover, this study also analyzed the levels of autophagy markers, LC3 and P62, in the pancreas and spleen [44]. Additionally, the immune-related signaling molecules can regulate autophagy, which is essential to improving immune disorders and the inflammatory response [48]. In recent years, scientists have proposed that autophagy disorder of islets of T1D patients, and autophagy can reduce the harmful effects of ER stress and DNA damage by delaying apoptosis of *β*-cells [49, 50]. A study about metformin reported that increasing autophagy can improve Th17 inflammation [51]. This study discovered that Rg1 stimulated the expression of LC3 and P62 proteins suggesting that Rg1 has a promoting effect on the autophagy of *β*-cells and spleen tissues. This report found that the effect of Rg1 on autophagy in T1D mice may be related to CXCL16 and NF-*κ*B. A study reported that the incretion of ox-LDL sensitized adipocytes to the lower insulin-induced glucose uptake and increased the levels of NF-*κ*B and the marker of apoptosis and autophagy (Bnip3), suggesting the ox-LDL was related to inflammation, apoptosis, and autophagy [52]. The effect of Rg1 on autophagy in T1D deserves further study. Additionally, this study has some shortcomings. If multiple dose groups of Rg1 can be set, the results would be more convincing in this study, and this study will further explore the effects of multi-dose Rg1 in the future. In summary, this paper studied the pharmacology efficacy of the mechanism of Rg1 improving T1D, which indicated that the protective impact of Rg1 on T1D might be associated with antagonizing inflammation and improving autophagy disorder. This team has planned to further explorate the relevant targets in the follow-up research.

## 5. Conclusion

The study successfully constructed T1D mice and revealed the biological action of Rg1 on improving T1D by antagonizing inflammation and improving autophagy disorders. It proved that the Rg1 ameliorated the blood glucose, body weight, pancreas histological damage. Also, this study proved that Rg1 decreased the INS level in serum and pancreas, and reduced the levels of CD45, CXCL16, INS, ox-LDL, and TF in the spleen and pancreas. And the inhibition of Rg1 on the mRNA levels of IL-1*β*, NOS2, and TNF-*α* in the spleen and pancreas suggested that Rg1 ameliorates T1D of mice by inhibiting inflammation. Interestingly, this study proved that the Rg1 treatment on T1D mice could raise the ratio of LC3 II protein to LC3 I protein which suggesting that Rg1 has potential to advance the autophagy to treatment of T1D. This study provides a scientific basis for the Rg1 treatment for T1D and a new direction for investigation of its biological mechanism.

## Figures and Tables

**Table 1:** Primer sequences.

Gene	Forward primer (5′-3′)	Reverse primer (5′-3′)
IL-1*β*	TTGAAGTTGACGGACCCCAA	TGTCCTGACCACTGTTGTTTC
TNF-*α*	TCACTGGAGCCTCGAATGTC	TCTGTGAGAAGGCTGTGCA
NOS2	CTGCAAGCACAATGGGGAGT	CGTCGGTAGAGAGACTGCTG
GAPDH	CGAGACACGATGGTGAAGGT	TGCCGTGGGTGGAATCATAC

## Data Availability

The data supporting the findings of this study are available from the corresponding author, G. X., upon reasonable request.
